# Joint Demosaicing and Denoising Based on Interchannel Nonlocal Mean Weighted Moving Least Squares Method

**DOI:** 10.3390/s20174697

**Published:** 2020-08-20

**Authors:** Yeahwon Kim, Hohyung Ryu, Sunmi Lee, Yeon Ju Lee

**Affiliations:** 1 Department of Mathematics, Graduate School, Kyung Hee University, Seoul 02447, Korea; myshu0619@khu.ac.kr (Y.K.); rootfna@khu.ac.kr (H.R.); 2 Department of Applied Mathematics, Kyung Hee University, Yongin 446-701, Korea; sunmilee@khu.ac.kr; 3 Division of Applied Mathematics, Korea University, Sejong 30019, Korea

**Keywords:** moving least squares approximation, demosaicing, patch similarity, color filter array

## Abstract

Nowadays, the sizes of pixel sensors in digital cameras are decreasing as the resolution of the image sensor increases. Due to the decreased size, the pixel sensors receive less light energy, which makes it more sensitive to thermal noise. Even a small amount of noise in the color filter array (CFA) can have a significant effect on the reconstruction of the color image, as two-thirds of the missing data would have to be reconstructed from noisy data; because of this, direct denoising would need to be performed on the raw CFA to obtain a high-resolution color image. In this paper, we propose an interchannel nonlocal weighted moving least square method for the noise removal of the raw CFA. The proposed method is our first attempt of applying a two dimensional (2-D) polynomial approximation to denoising the CFA. Previous works make use of 2-D linear or directional 1-D polynomial approximations. The reason that 2-D polynomial approximation methods have not been applied to this problem is the difficulty of the weight control in the 2-D polynomial approximation method, as a small amount of noise can have a large effect on the approximated 2-D shape. This makes CFA denoising more important, as the approximated 2-D shape has to be reconstructed from only one-third of the original data. To address this problem, we propose a method that reconstructs the approximated 2-D shapes corresponding to the RGB color channels based on the measure of the similarities of the patches directly on the CFA. By doing so, the interchannel information is incorporated into the denoising scheme, which results in a well-controlled and higher order of polynomial approximation of the color channels. Compared to other nonlocal-mean-based denoising methods, the proposed method uses an extra reproducing constraint, which guarantees a certain degree of the approximation order; therefore, the proposed method can reduce the number of false reconstruction artifacts that often occur in nonlocal-mean-based denoising methods. Experimental results demonstrate the performance of the proposed algorithm.

## 1. Introduction

Demosaicing refers to the task of reconstructing a full-color image from incomplete color samples sensed by the image sensor of a digital camera. The color filter array (CFA), which is placed over the image sensor, determines what colored pixels are missing. The most commonly used color filter array is the Bayer CFA [1]. One significant problem for the demosaicing process is the problem of noise. Nowadays, the sizes of the pixel sensors are decreasing with the increase in the resolutions of camera sensors. This makes the pixel sensors more sensitive to noise as the smaller sensors often receive less light energy.

Conventionally, the denoising process was applied as a postprocessing effect after the demosaicing. This is because the correlation between neighboring pixels is larger in the demosaiced image than in the raw CFA image, which makes it easier to apply a local denoising process. For example, in [2], a polynomial interpolation-based demosaicing method was proposed to resolve both the noise and the zippering and false color artifacts that occur on object boundaries by making some contributions associated with the calculation of error predictors and edge classification. The well-known nonlocal-mean-based denoising scheme was introduced into the demosaicing problem in [3]. Nonlocal mean denoising is a self-similarity driven method that incorporates the interaction between colors and the local image geometry, and was shown to remove the noise effectively. Another way to retain the sharp edges during the demosaicing process is the interpolation of the residuals, i.e., the differences between the original and the tentative images [4]. In this approach, first, the tentative image is estimated by an edge-preserving guided filter [5]; then, the estimated residual images are interpolated by some demosaicing algorithms, e.g., the gradient-based threshold-free method [6].

A major problem when denoising is applied as a postprocess to the demosaicing is that the effect of noise on the demosaicing process cannot be undone after the demosaicing. Even when there is only a small amount of noise in the color filter array, the effect on the demosaiced image can still be significant as the noise affects the reconstruction of two-thirds of the missing data. Because of this, it has become a recent trend to denoise the raw CFA image before applying the demosaicing process [7,8,9]. Applying denoising as a preprocess is difficult because denoising the raw CFA is a more challenging task than denoising the demosaiced image as two-thirds of the data are missing. The distances between the data points are larger than in the demosaiced image. The authors of [7] try to first augment the insufficient data in the raw CFA by converting the raw CFA data to a pseudo-four-channel image (two green, red, and blue channels). The four-channel data are transformed based on principal component analysis (PCA). Since the signal energy is compact while the noise is uniformly distributed, a block-matching and 3D filtering method (BM3D) works very well for denoising in the transformed space. Lastly, the denoised data are rearranged to obtain the denoised CFA raw data and the residual interpolation (RI) method is used for demosaicing. The authors of [8] utilize cross-color correlations into a modified block-matching 3D-filter-based (BM3D) denoising, while the authors of [9] use a unified objective function which incorporates the BM3D filtering, the total variation minimization efficiently by the alternating direction method of multipliers (ADMM). Besides the traditional approach in demosaicing, there are attempts to achieve joint demosaicing and denoising by using convolutional neural networks (CNNs) such as the works of [10,11,12,13,14]. Besides normal CNNs, a generative adversarial network (GAN)-based method [15] and fully end-to-end deep neural models [16,17] have also been proposed to tackle the demosaicing problem. However, the drawback with using neural networks is that large amounts of data are required to train the network. Therefore, even though the performance of some CNNs can achieve state-of-the-art results, it is still important to develop methods that can work with a single The works of [18,19] propose the use of white pixels obtained by an RGBW color filter array and observed that white pixels are helpful for demosaicing in low-light conditions. The work of [20] realized that intracolor demosaicing is important to reduce the color artifacts, while the work of [21] analyzed the real color acquisition process from the raw image to the sRGB image space and showed that it is proper to perform a denoising process directly on the raw sensor data. Mathematical tools like anisotropic diffusion [22] or polarization-based [23] methods have also been applied to the problem of demosaicing; the work in [22] developed a linear anisotropic diffusion method for any arbitrary kind of color filter arrays, while the work in [23] applied an extension of the CFA to a polarization filter array (PFA). In this paper, we apply an approximation-based method for the problem of demosaicing. Approximation-based denoising methods have been applied in various fields. The work in [24] applied a nonlinear moving least-squares projection method for the denoising of high-dimensional noisy scattered data, while the work in [25] applied a biquadratic polynomial approximation for denoising of medical images. In [26], a modified singular value thresholding with minimizing error constraints is used for the segmentation of noisy signals by an orthogonal polynomial approximation.

In this paper, we propose a nonlocal interchannel weighted moving least square method for the denoising of noisy Bayer CFAs. The proposed method is a first attempt of applying a two-dimensional polynomial approximation-based denoising on noisy CFA patterns. Previous conventional joint denoising and demosaicing methods take either a 2-D approach with nonpolynomial approximation, e.g., a two-dimensional linear weighted sum of the neighboring pixels, or a directional 1-D polynomial approximation approach. The reason that previous polynomial approximation methods do not use a 2-D approach is due to the difficulty of the weight control in forming the 2-D approximation shape. If the conventional weighted least squares approach is applied to the 2-D polynomial approximation, the resulting 2-D approximation shape cannot represent the discontinuities of edge regions well, and therefore, will not result in a smooth image. To overcome this problem in our method, we compute the nonlocal mean weights for the weighted least square directly from the raw color filter array by taking the interchannel information into account. The goal of solving the weighted least squares problem is to find the filter coefficients for the 2-D filters that are used in the reconstruction of the pixel values; the better weights will result in finding better 2-D filters. After the filter coefficients are obtained, we again perform a nonlinear function on these coefficients so that the filtering of the data points with the 2-D filters only involves the important data points.

Compared with nonlocal-mean-based methods, e.g., the BM3D denoising, which only uses the mean filtering based on the nonlocal similarities of the pixels, the proposed method incorporates an extra reproducing constraint into the denoising scheme. Normally, nonlocal-mean-based denoising methods work well when there are iterative patterns in the image; however, if the correlations between the patches in the search region weaken, nonlocal-mean-based denoising methods can produce many false intensity values. This is because the denoising is performed without considering the approximation error. In comparison, the reproducing constraint is directly related to the reconstruction error, i.e., the approximation error. Using a reproducing constraint is better than nonlocal-mean-based methods in that it prevents the approximated denoised CFA image from being too different from the original CFA image. This improves the approximation accuracy.

We summarize the main contributions of the proposed method as follows:

- For the first time, we applied a two-dimensional polynomial-approximation-based denoising on noisy CFA patterns.
- Compared with nonlocal-mean-based methods, e,g., the BM3D denoising, the proposed method incorporates an extra reproducing constraint into the denoising scheme. This guarantees an approximation accuracy to a desired order.
- We incorporate interchannel information into the polynomial approximation by determining the nonlocal weights directly from the noisy raw CFA image.

## 2. Relation of the Proposed Work to Sensors

**Figure 1:**
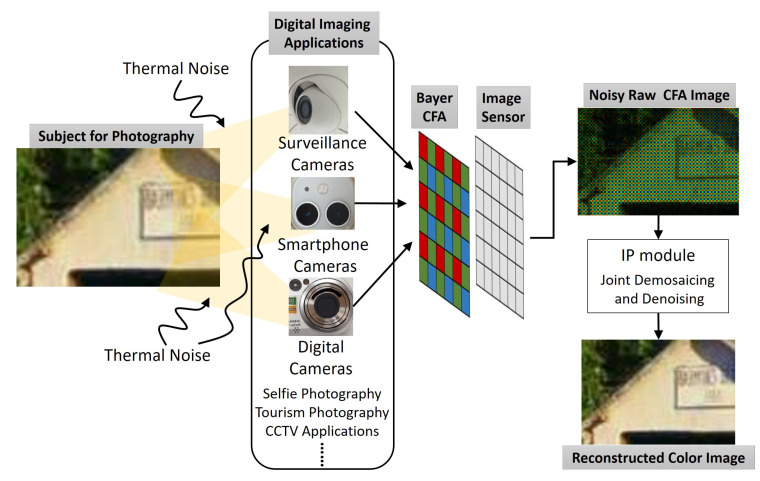
Acquisition system of digital cameras in digital imaging applications. CFA—color filter array, IP—image processing.

Nowadays, most digital cameras acquire images with a single monochrome image sensor overlaid by a color filter array (CFA) to capture color information (Figure 1). The reason for the use of a single image sensor in digital cameras is to reduce the cost. However, due to the use of a single image sensor, the color channels are undersampled and the missing color information has to be restored. The aim of a demosaicing algorithm in the IP (Image Processing) module in digital cameras is to reconstruct the full color image from the spatially undersampled color channels output from the CFA. As the original CFA image is noisy and of low resolution, demosaicing algorithms are centered on resolution improvement and denoising. This paper proposes a joint denoising and demosaicing framework based on the moving least square (MLS) method and, therefore, helps to overcome the physical limitations of single monochrome image sensors in digital cameras.

## 3. Related Works

### 3.1. Residual Interpolation

The residual interpolation (RI) method proposed in [4] is a demosaicing method with excellent performance. It is an algorithm developed by integrating the residual interpolation method with the gradient-based threshold-free (GBTF) algorithm [6]. With this method, first, a tentative G pixel value is estimated at the positions of the R and B pixels by the Hamilton and Adams interpolation as in the GBTF algorithm. After that, the R and B pixel values are interpolated by the residual interpolation process. For example, for the R image, the RI algorithm first generates the tentative estimate ($\tilde{R}$) of the R image by a guided upsampling process, and then estimates the residuals between the *R* pixel and the $\tilde{R}$ pixel values at the R pixel positions. The residuals are also interpolated to result in the interpolated residual image. After that, the demosaiced R image $\hat{R}$ is constructed by adding $\tilde{R}$ to the interpolated residual image. The demosaiced B image $\hat{B}$ is constructed in the same manner. In the proposed method, we utilize the RI method to construct the full-color image after the denoised CFA image has been obtained with the proposed interchannel nonlocal mean weighted moving least squares method.

### 3.2. Moving Least Square Methods with Total Variation Minimization

Moving least squares refers to the method of reconstructing a continuous function from a set of unorganized point samples by calculating a weighted least squares measure around the point at which the reconstruction is required. The solution of the MLS method has a closed-form and is easily computed by solving a linear system. It was shown to be quite useful in interpolation-based image-processing such as super-resolution and image zooming [27,28,29]. The MLS method has been applied to 2-D linear and nonlinear systems [30] for the interpolation in meshless environments [31] and for other image processing tasks such as nonlinear color transfer [32], but not to the problem of denoising the CFA image, since one of the major drawbacks of the MLS method is that it is weak against the noise. To overcome the problem of weakness against noise, in [33], we proposed the incorporation of the total variation regularization [34] into the MLS framework for better denoising power; however, if the MLS formulation in [33] is directly applied to the joint demosaicing and denoising problem, it yields poor results, as the correlation between the color channels cannot be well measured because two-thirds of the data are missing and the positions of the missing data are different for each color channel. Therefore, to apply the MLS method to denoise the color filter array, the formulation has to be changed to fit the problem and the weights have to be calculated while taking the interchannel information into account.

## 4. Proposed Method

### 4.1. Problem Formulation

Let the ground-truth color image on an image domain Ω be given as $u={\left\{u\left(i,j\right)\right\}}_{i,j\in \Omega }$ with $u\left(i,j\right)={\left[R\left(i,j\right),G\left(i,j\right),B\left(i,j\right)\right]}^{T}$. The problem of demosaicing is to construct a color image $u^{*}$ from a noisy CFA, *x*, where *x* is the addition of the mosaiced pattern image *u* and the noise *n*:

x(i,j)=u(i,j)+n(i,j),(i,j)∈Ω.

It should be noted that u is a color image, which is why we use a vector representation for u, while the mosaiced pattern image *u* is a monotone CFA image. In this paper, the mosaiced pattern image *u* is a Bayer pattern image and *n* is a white Gaussian noise which follows a normal distribution $n\left(i,j\right)∼N\left(0,\sigma^{2}\right)$ with a certain variance σ. Our goal is to reconstruct $u^{*}$ as close as possible to the true image u. For the denoising of the CFA, we first construct a local polynomial approximation function for each pixel that well reflects the local structure at that pixel. We then take the coefficients of the local polynomial approximation function and apply a nonlinear transform on these coefficients. After that, we take the dot product of the filter with a neighborhood of pixels to decide the value of the centered pixel. The filtered pixels become the denoised version of the original noisy pixels in the CFA. We describe the proposed method in detail in the following subsection.

### 4.2. Interchannel Data Weighted Least Squares Reconstruction

Let *x* be a given mosaiced CFA image and let $p_{C}=\left(p_{C},q_{C}\right)$ denote a pixel position corresponding to the color C∈{R,G,B}. We want to construct the local polynomial approximation functions $L_{p_{C}}\left(r\right)$ corresponding to each color C∈{R,G,B}:

$$L_{p_{C}}\left(r\right):=\sum_{{\left|\alpha \right|}_{1}\leq m}c_{\alpha }r^{\alpha },\;r\in \Omega_{p_{C}},$$

where $\Omega_{p_{C}}$ denotes the set of pixels that are in the neighborhood of $p_{C}$ and have the same color as $p_{C}$. For example, $\Omega_{p_{G}}:=\Omega_{p_{C=G}}$ contains only the Green pixels in the CFA that are in the neighborhood of $p_{C=G}$, i.e., $\Omega_{p_{G}}$ contains the nearest 41 green pixel positions (p,,q) which are $\left(p,q\right)=\left(p_{G}-2i,q_{G}-2j\right),i,j=-2,-1,0,1,2$ and $\left(p,q\right)=\left(p_{G}-2i-1,q_{G}-2j-1\right),i,j=-1,0,1,2$. Then, the polynomial $L_{p_{C}}$ is constructed to match the distribution of the data in the set $\Omega_{p_{C}}$. By doing so, $L_{p_{C}}$ takes into account the local structure at $p_{C}$. For the Red and Blue pixels in the CFA, we construct the polynomials $L_{p_{R}}$ and $L_{p_{B}}$ from the data of larger regions, i.e., from the nearest 49 Red pixels or Blue pixels, respectively. For a specific Red pixel $p_{R}=\left(p_{R},q_{R}\right)$, the nearest 49 Red pixels are $\left(p,q\right)=\left(p_{R}-2i,q_{R}-2j\right),i,j=-3,-2,-1,0,1,2,3$, while the nearest 49 Blue pixels for $p_{B}$ are $\left(p,q\right)=\left(p_{B}-2i,q_{B}-2j\right),i,j=-3,-2,-1,0,1,2,3$.

The polynomial $L_{p_{G}}\left(p_{G}\right)$ can be obtained by the following minimization, which takes the total variation regularization into account:

(1) $$\begin{array}{cc} \underset{L_{p_{G}}\in \Pi_{m}}{\mathrm{argmin}}\{ & \sum_{i,j=-2}^{2}\left(|\nabla L_{p_{G}}\left(p_{G}+2i,q_{G}+2j\right)|+\frac{\mu }{2}|L_{p_{G}}\left(p_{G}+2i,q_{G}+2j\right)-x\left(p_{G}+2i,q_{G}+2j\right){|}^{2}\theta_{p_{G}}\left(p_{G}+2i,q_{G}+2j\right)\right)\\ \; & +\sum_{i,j=-1}^{2}(|\nabla L_{p_{G}}\left(p_{G}+2i-1,q_{G}+2j-1\right)|\\ \; & +\frac{\mu }{2}|L_{p_{G}}\left(p_{G}+2i-1,q_{G}+2j-1\right)-x\left(p_{G}+2i-1,q_{G}+2j-1\right){|}^{2}\theta_{p_{G}}\left(p_{G}+2i-1,q_{G}+2j-1\right))\}. \end{array}$$

**Figure 2:**
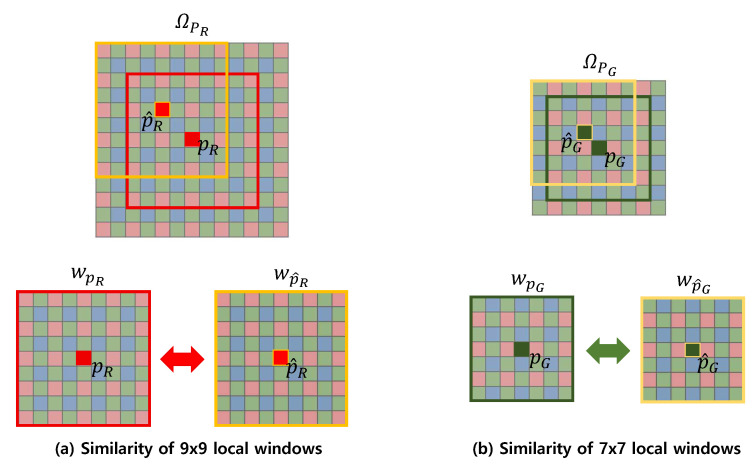
Measuring the local structure similarity using (**a**) 9×9 windows for R and B pixels and (**b**) 7×7 windows for G pixels.

It should be noticed that the gradients $\nabla L_{p_{G}}\left(p_{G}+2i,q_{G}+2j\right)$ and $\nabla L_{p_{G}}\left(p_{G}+2i-1,q_{G}+2j-1\right)$ are computed by taking only the Green pixels in the CFA into account and not the neighboring pixels, as in the case of computing the gradients in normal images. For the computation of $L_{p_{R}}\left(p_{R}\right)$ and $L_{p_{B}}\left(p_{B}\right)$, we solve the following minimization problems,

(2) $$\underset{L_{p_{R}}\in \Pi_{m}}{\mathrm{argmin}}\left\{\sum_{i,j=-3}^{3}\left(|\nabla L_{p_{R}}\left(p_{R}+2i,q_{R}+2j\right)|+\frac{\mu }{2}{\left|L_{p_{R}}\left(p_{R}+2i,q_{R}+2j\right)-x\left(p_{R}+2i,q_{R}+2j\right)\right|}^{2}\theta_{p_{R}}\left(p_{R}+2i,q_{R}+2j\right)\right)\right\},$$

and

(3) $$\underset{L_{p_{B}}\in \Pi_{m}}{\mathrm{argmin}}\left\{\sum_{i,j=-3}^{3}\left(|\nabla L_{p_{B}}\left(p_{B}+2i,q_{B}+2j\right)|+\frac{\mu }{2}{\left|L_{p_{B}}\left(p_{B}+2i,q_{B}+2j\right)-x\left(p_{B}+2i,q_{B}+2j\right)\right|}^{2}\theta_{p_{B}}\left(p_{B}+2i,q_{B}+2j\right)\right)\right\},$$

where once again the gradients $\nabla L_{p_{R}}\left(p_{R}+2i,q_{R}+2j\right)$ and $\nabla L_{p_{B}}\left(p_{B}+2i,q_{B}+2j\right)$ are computed with respect to the Red pixels and Blue pixels in the CFA, respectively. The weighting functions $\theta_{p_{C}},C\in \left\{R,G,B\right\}$ are defined as

$$\begin{array}{c} \theta_{p_{G}}\left({\hat{p}}_{G},{\hat{q}}_{G}\right):=\exp\left\{-\frac{1}{h_{0}^{2}}\sum_{i,j=-3}^{3}G_{\sigma }\left(i,j\right){\left(x\left(p_{G}+i,q_{G}+j\right)-x\left({\hat{p}}_{G}+i,{\hat{q}}_{G}+j\right)\right)}^{2}\right\}, \end{array}$$

for Equation (1), and

$$\begin{array}{c} \theta_{p_{R}}\left({\hat{p}}_{R},{\hat{q}}_{R}\right):=\exp\left\{-\frac{1}{h_{0}^{2}}\sum_{i,j=-4}^{4}G_{\sigma }\left(i,j\right){\left(x\left(p_{R}+i,q_{R}+j\right)-x\left({\hat{p}}_{R}+i,{\hat{q}}_{R}+j\right)\right)}^{2}\right\}, \end{array}$$

$$\begin{array}{c} \theta_{p_{B}}\left({\hat{p}}_{B},{\hat{q}}_{B}\right):=\exp\left\{-\frac{1}{h_{0}^{2}}\sum_{i,j=-4}^{4}G_{\sigma }\left(i,j\right){\left(x\left(p_{B}+i,q_{B}+j\right)-x\left({\hat{p}}_{B}+i,{\hat{q}}_{B}+j\right)\right)}^{2}\right\}, \end{array}$$

for Equations (2) and (3), respectively, where $G_{\sigma }\left(\cdot ,\cdot \right)$ denotes a Gaussian function with standard deviation σ. The nonlocal weight $\theta_{p_{C}}$ measures the similarity of the data structure of $p_{C}=\left(p_{C},q_{C}\right)$ and ${\hat{p}}_{C}=\left({\hat{p}}_{C},{\hat{q}}_{C}\right)$, where $p_{C}=\left(p_{C},q_{C}\right)$ and ${\hat{p}}_{C}=\left({\hat{p}}_{C},{\hat{q}}_{C}\right)$ are the pixels that belong to the same color; however, even though $\theta_{p_{C}}$ measures the similarity between pixels of the same color, all the RGB pixels are involved in the computation of the similarity. For example, even though ${\hat{p}}_{G}=\left({\hat{p}}_{G},{\hat{q}}_{G}\right)$ is the position of the pixel belonging to the Green color, $\left(i,j\right),\;i={\hat{p}}_{G}-3,\cdots ,{\hat{p}}_{G}+3,j={\hat{q}}_{G}-3,\cdots ,{\hat{q}}_{G}+3$ are the pixel positions of all colors. This is in contrast with (1)–(3), where we used only the pixels corresponding to a specific color. The incorporation of all the RGB pixels provides the computation of the similarity with interchannel information. This enhances the accuracy in computing the weighting functions. As a different weighting function results in a different local polynomial approximation function, interchannel information results in a better polynomial approximation. The size of the window that contains the pixels involved in the computation is 7×7 for the computation of $\theta_{p_{G}}$ and 9×9 for the computation of $\theta_{p_{R}}$ and $\theta_{p_{B}}$, as shown in Figure 2. Normally, after obtaining all the local polynomial approximation functions $L_{p_{C}}\left(r\right)$ corresponding to all colors C∈{R,G,B} and all pixels r, we already can obtain the denoised CFA image. This can be done by evaluating the values of all the functions $L_{p_{C}}$ at the points where the functions have been constructed, i.e., evaluate the values $L_{p_{C}}\left(p_{C}\right)$ for all $p_{C}$. By integrating all the values $L_{p_{C}}\left(p_{C}\right)$ into a 2-D image, we obtain the denoised CFA image; however, with the proposed method, we take one more step to eliminate the effect of the data points that contribute little to the reconstruction of the function values $L_{p_{C}}\left(p_{C}\right)$. This can be done by first utilizing the theorem that the $L_{p_{C}}\left(p_{C}\right)$ can be expressed as a dot product between the filter weights and the data points [35]. Applying the theorem to our case, we can rewrite the function values $L_{p_{G}}\left(p_{G}\right)$, $L_{p_{R}}\left(p_{R}\right)$, and $L_{p_{B}}\left(p_{B}\right)$ as

(4) $$L_{p_{G}}\left(p_{G}\right):=\sum_{i,j=-2}^{2}w_{2i,2j}^{G}x\left(p_{G}+2i,q_{G}+2j\right)+\sum_{i,j=-1}^{2}w_{2i-1,2j-1}^{G}x\left(p_{G}+2i-1,q_{G}+2j-1\right),$$

(5) $$L_{p_{R}}\left(p_{R}\right):=\sum_{i,j=-3}^{3}w_{i,j}^{R}x\left(p_{R}+2i,q_{R}+2j\right),$$

(6) $$L_{p_{B}}\left(p_{B}\right):=\sum_{i,j=-3}^{3}w_{i,j}^{B}x\left(p_{B}+2i,q_{B}+2j\right).$$

By inserting (4)–(6) into (1)–(3), respectively, we can obtain the filter coefficients $\left\{w_{2i,2j}^{G}\right\}$, $\left\{w_{2i-1,2j-1}^{G}\right\}$, $\left\{w_{i,j}^{R}\right\}$, and $\left\{w_{i,j}^{B}\right\}$. Next, we apply nonlinear transforms on the filter coefficients to get

(7) $$\phi_{th}^{even}\left(w_{2i,2j}^{G}\right)=\frac{1_{|w_{2i,2j}^{G}|\geq th}w_{2i,2j}^{G}}{\sum_{i,j=-2}^{2}1_{|w_{2i,2j}^{G}|\geq th}+\sum_{i,j=-1}^{2}1_{|w_{2i-1,2j-1}^{G}|\geq th}},$$

(8) $$\phi_{th}^{odd}\left(w_{2i-1,2j-1}^{G}\right)=\frac{1_{|w_{2i-1,2j-1}^{G}|\geq th}w_{2i-1,2j-1}^{G}}{\sum_{i,j=-2}^{2}1_{|w_{2i,2j}^{G}|\geq th}+\sum_{i,j=-1}^{2}1_{|w_{2i-1,2j-1}^{G}|\geq th}},$$

(9) $$\phi_{th}\left(w_{i,j}^{R}\right)=\frac{1_{|w_{i,j}^{R}|\geq th}w_{i,j}^{R}}{\sum_{i,j=-3}^{3}1_{|w_{i,j}^{R}|\geq th}},$$

(10) $$\phi_{th}\left(w_{i,j}^{B}\right)=\frac{1_{|w_{i,j}^{B}|\geq th}w_{i,j}^{B}}{\sum_{i,j=-3}^{3}1_{|w_{i,j}^{B}|\geq th}},$$

where

$$1_{|w_{a,b}^{C}|\geq th}=\left\{\begin{array}{cc} 1 & \mathrm{if}\;|w_{a,b}^{C}|\geq th\\ 0 & \mathrm{if}\;|w_{a,b}^{C}|<th \end{array}\right.\;\;\;for\;\;C=R,G,B.$$

The above transform filters out the filter coefficients that are smaller than a predefined threshold value th, and renormalize the remaining filter coefficients. By filtering out the small filter coefficients, the data points that have little contribution to the construction of $L_{p_{C}}\left(p_{C}\right)$ are excluded from the construction process. This prevents unrelated pixels from affecting the construction process, which prevents an oversmoothed CFA.

Now, using the transformed filter coefficients $\left\{\phi_{th}^{even}\left(w_{2i,2j}^{G}\right)\right\}$, $\left\{\phi_{th}^{odd}\left(w_{2i-1,2j-1}^{G}\right)\right\}$, $\left\{\phi_{th}\left(w_{i,j}^{R}\right)\right\}$, and $\left\{\phi_{th}\left(w_{i,j}^{B}\right)\right\}$ instead of the original weights, we construct ${\tilde{L}}_{p_{C}}\left(p_{C}\right),{\;}_{C=R,G,B}$ instead of $L_{p_{C}}\left(p_{C}\right),{\;}_{C=R,G,B}$:

(11) $${\tilde{L}}_{p_{G}}\left(p_{G}\right):=\sum_{i,j=-2}^{2}\phi_{th}^{even}\left(w_{2i,2j}^{G}\right)x\left(p_{G}+2i,q_{G}+2j\right)+\sum_{i,j=-1}^{2}\phi_{th}^{odd}\left(w_{2i-1,2j-1}^{G}\right)x\left(p_{G}+2i-1,q_{G}+2j-1\right),$$

(12) $${\tilde{L}}_{p_{R}}\left(p_{R}\right):=\sum_{i,j=-3}^{3}\phi_{th}\left(w_{i,j}^{R}\right)x\left(p_{R}+2i,q_{R}+2j\right),$$

(13) $${\tilde{L}}_{p_{B}}\left(p_{B}\right):=\sum_{i,j=-3}^{3}\phi_{th}\left(w_{i,j}^{B}\right)x\left(p_{B}+2i,q_{B}+2j\right).$$

**Figure 3:**
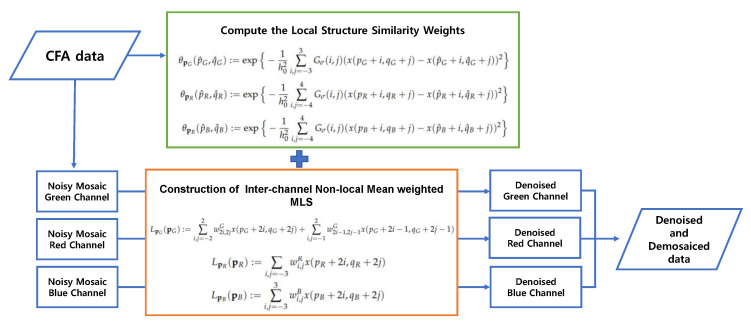
Block diagram of the proposed algorithm. MLS—moving least square.

**Figure 4:**
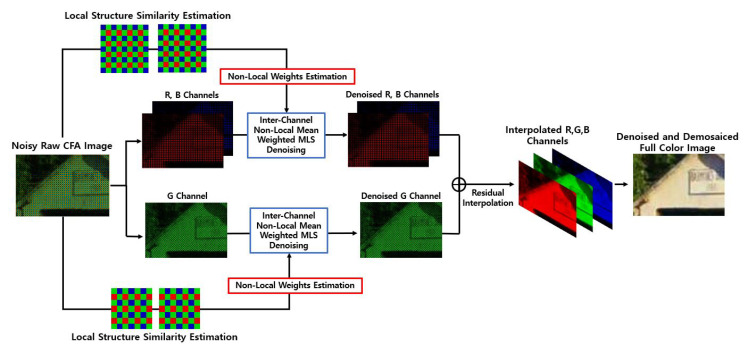
Flow Process of the proposed algorithm.

Finally, we replace all the intensity values in ${\left\{u\left(p_{C},q_{C}\right)\right\}}_{p_{C},q_{C}\in \Omega ,\;C=R,G,B}$ in the noisy CFA with ${\left\{{\tilde{L}}_{p_{C}}\left(p_{C},q_{C}\right)\right\}}_{p_{C},q_{C}\in \Omega ,\;C=R,G,B}$, where Ω is the domain corresponding to the CFA image. Here, $\left(p_{C},q_{C}\right)$ refers to the pixel position corresponding to the color *C*. With the denoised CFA ${\left\{{\tilde{L}}_{p_{C}}\left(p_{C},q_{C}\right)\right\}}_{p_{C},q_{C}\in \Omega ,\;C=R,G,B}$, we are now ready to apply the residual interpolation algorithm [4] to obtain the full-color image, i.e., the three-channel array $u^{*}={\left\{u^{*}\left(i,j\right)\right\}}_{i,j\in \Omega }$, where $u^{*}\left(i,j\right)={\left[R^{*}\left(i,j\right),G^{*}\left(i,j\right),B^{*}\left(i,j\right)\right]}^{T}$ is the full-color pixel reconstructed by the demosaicing method. Figure 3 shows the block diagram of the proposed joint denoising and demosaicing method, while Figure 4 shows how the input image is processed by the proposed method and also shows the intermediate image results at each stage.

## 5. Experimental Results

We compared the joint denoising and demosaicing results of the proposed scheme with the RI [4] to show the denoising power of the proposed scheme—the block-matching 3D (BM3D) filtering [7], which uses an enhanced sparse representation in transform-domain; and the alternating direction minimization multipliers (ADMM) method [9], which efficiently incorporates the total variation minimization and the BM3D filtering into a unified framework. The BM3D and the ADMM methods are state-of-the-art methods for denoising the color filter array. Both the ADMM and the BM3D use a denoising method that groups similar patches into a 3D volume, then performs a transform-domain shrinkage on this 3D volume. The collaborative filtering used in these methods can reveal even the finest details shared by grouped blocks, and therefore, can preserve essential and unique features of each block.

The experiments show that the proposed method is comparable to the state-of-the-art denoising methods and can better preserve some features that the ADMM and BM3D methods cannot. We experimented on the McMaster dataset, which contains images that are closer to the images taken with a real digital camera than the Kodak dataset. We used two noisy datasets of different noise levels, i.e., on a dataset with weak noise where the noise is generated from a zero-mean Gaussian distribution with σ = 7.65, and a dataset with a higher level of noise generated with σ = 12.75, where σ is the standard deviation.

**Figure 5:**
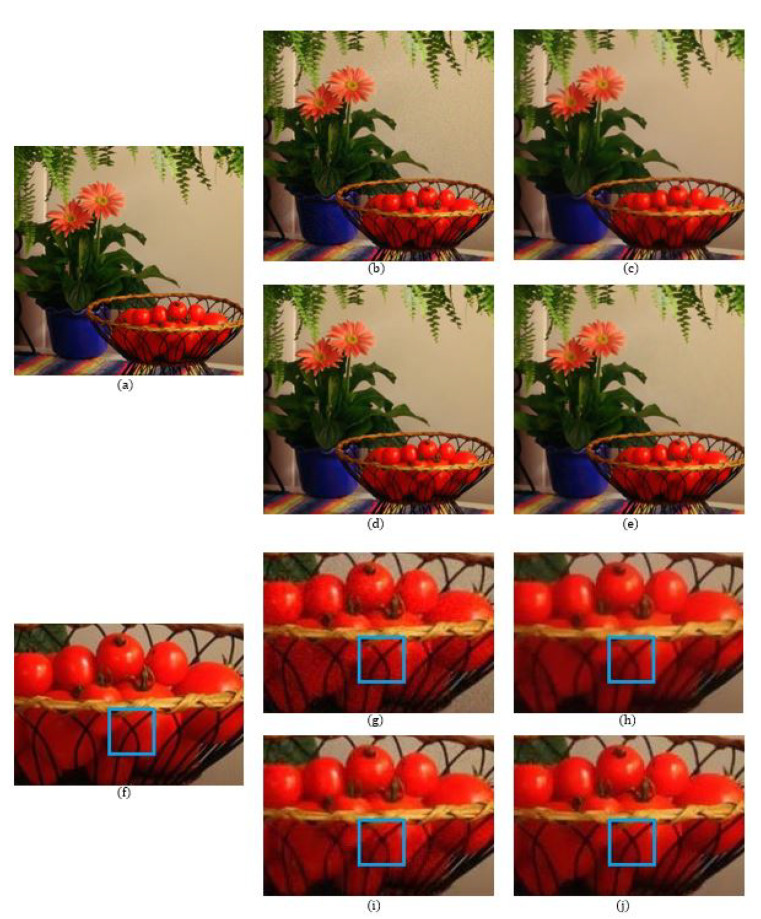
Comparison of the results between the different methods on MCM image 9: (**a**,**f**) Original; (**b**,**g**) residual interpolation (RI) [4] (psnr = 30.3286 dB); (**c**,**h**) alternating direction minimization multipliers (ADMM) [9] (psnr = 29.2356 dB); (**d**,**i**) block-matching and 3D filtering method (BM3D) [7] (psnr = 30.9309 dB); (**e**,**j**) proposed (psnr = 30.5131 dB).

**Figure 6:**
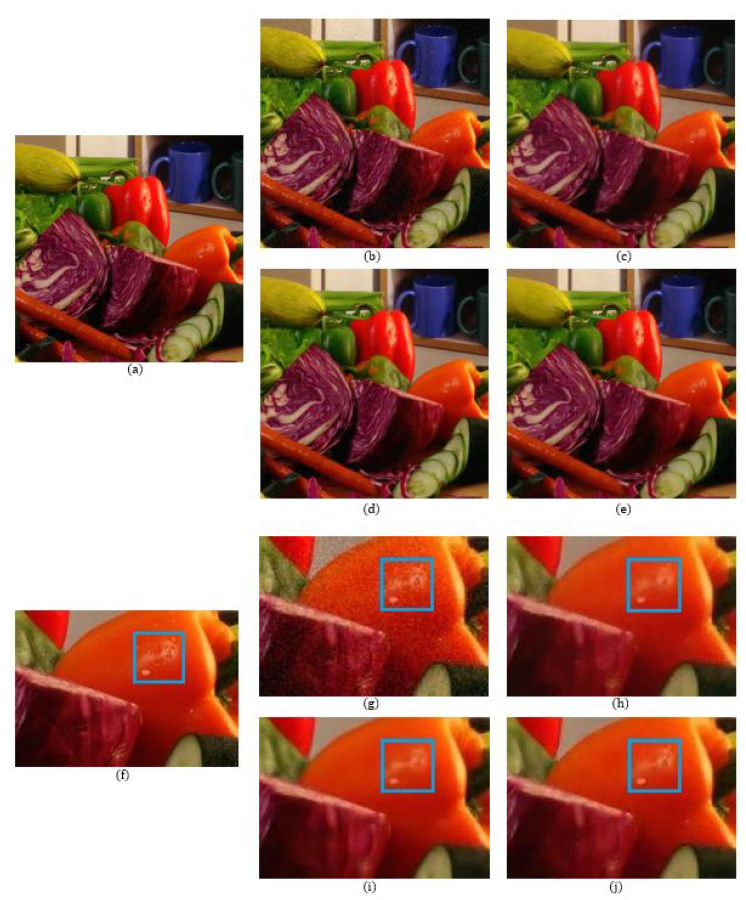
Comparison of the results between the different methods on MCM image 10: (**a**,**f**) Original; (**b**,**g**) RI [4] (psnr = 29.2452 dB); (**c**,**h**) ADMM [9] (psnr = 29.8837 dB); (**d**,**i**) BM3D [7] (psnr = 30.9424 dB); (**e**,**j**) proposed (psnr = 30.7439 dB).

**Figure 7:**
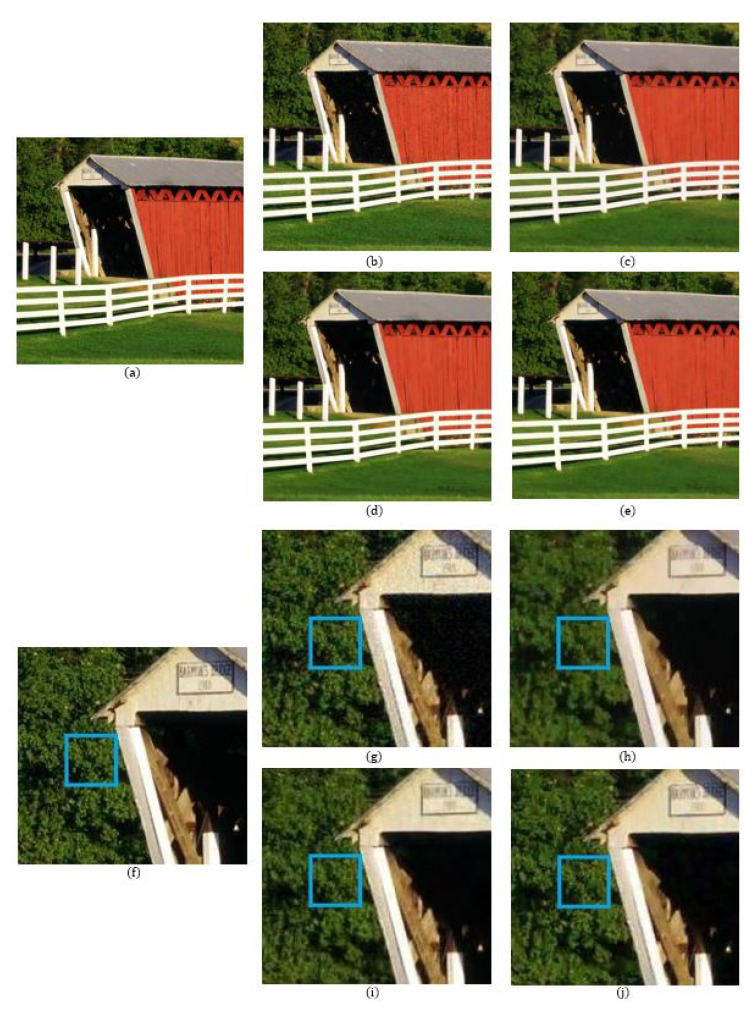
Comparison of the results between the different methods on MCM image 18: (**a**,**f**) Original; (**b**,**g**) RI [4] (psnr = 27.7879 dB); (**c**,**h**) ADMM [9] (psnr = 28.2356 dB); (**d**,**i**) BM3D [7] (psnr = 29.2443 dB); (**e**,**j**) proposed (psnr = 28.6549 dB).

The number of data points used in the reconstructions of the local polynomial approximation functions in the Green channel is 41, where the data are extracted from a 9×9 window. For the Red and the Blue channels, the number of data points is 49 for each channel, which is extracted from a 15×15 region. The degree of polynomials and the width of the Gaussian for the Green channel are 3 and 0.3, respectively, while for the Red and Blue channels, the degree of polynomials and the width of the Gaussian are both 3 and 0.5, respectively. Figure 5 shows a comparison of the denoising results on the MCM 9 image with weak noise (σ = 7.65). We see that even though the BM3D method has the highest PSNR value, there are some blurry artifacts, which can be observed in the enlarged images of Figure 5 and especially in the region corresponding to the fruit basket frame, as can be seen in Figure 5i. The reason that the BM3D results in blurry artifacts is due to the fact that the fruit basket image has little repeatable patterns, therefore, the 3D filtering in the BM3D blurs the thin frame regions. This is also true for the ADMM method, as can observed in Figure 5h. However, with the proposed method, the fruit basket frame is better reconstructed and more distinctive, as can be seen in Figure 5j, which is due to the fact that the proposed method applies the reproducing constraint in the reconstruction process. Table 1, Table 2 and Table 3 compare the PSNR, FSIM, and SSIM values between the different demosaicing methods. The tables show the PSNR, FSIM, and SSIM values for all 18 images in the McMaster dataset for different noise levels and different demosaicing methods. The proposed method is comparable to the state-of-the-art joint demosaicing and denoising methods. The BM3D method in [7] shows the largest PSNR, FSIM, or SSIM values for most images due to the large denoising power, but also shows some local artifacts when there is no repeatable pattern, or when the image structure is small. In this case, the proposed method shows more desirable results. This can be observed again in the dataset with more noise (σ = 12.75). Figure 6 and Figure 7 show the results of the MCM 10 and 18 images. Again, it can be observed in the enlarged images of the ADMM (Figure 6h) and the BM3D (Figure 6i) methods that the small details become blurry, which can be observed especially inside the blue boxes. However, the small details are sharply reconstructed with the proposed method, as can be seen in Figure 6j. Figure 7 shows that the colors of the small-scaled structures are better preserved with the proposed method. It can be observed in the enlarged images of the ADMM (Figure 7h) and the BM3D (Figure 7i) methods that the brown color has been diffused to green, especially inside the blue boxes, whereas in the original image (Figure 7f) there are several small-scaled structures with brown colors. The small-scaled structures with brown colors are well preserved with the proposed method as can be seen in Figure 7j.

## 6. Conclusions

In this paper, we proposed a two-dimensional polynomial-approximation-based denoising method with nonlocal weights for the denoising of noisy color filter arrays (CFAs). It is difficult to construct a two-dimensional approximation function from the sparse data points in the CFA image that can represent the original color image well, since the missing information makes it difficult to extract the data points for the two-dimensional approximation that can reconstruct the full color image with high accuracy. The major contribution of this paper is the proposal of a method that can compute the weights that indicate the importance of data points with high accuracy, so that the data points that are close to the point at which the reconstructed value is requested can be extracted with high accuracy. To obtain a high-accuracy estimation of the data points and a high-accuracy two-dimensional polynomial approximation, we incorporated the interchannel information into the calculation of the similarity weights. This results in the reconstruction of digital color images with high visual quality. After the weights have been obtained, we applied a nonlinear transform on the filter coefficients and reconstructed the denoised CFA pixels. We also eliminated the data points that have little contribution to the construction of the local filters to exclude the unrelated pixels from the construction process, which prevents an oversmoothed CFA image. Due to the use of the interchannel nonlocal mean weights and the incorporation of the reproducing constraint and the transformed filtered coefficients, we could get an approximation function that effectively preserves the small-scaled features in the image that other conventional denoising schemes cannot preserve well. This is due to the fact that conventional denoising schemes do not possess the extra reproducing constraint, whereas with the proposed scheme, we could get a reproducing constraint with high order due to the highly accurate weights and data points. The high order of the proposed approximation method improves the resolution in the demosaiced image.

One of the advantages of the proposed method is that it can be easily extended to be used with other CFAs due to the characteristics of the MLS, which reconstructs a function from a set of unorganized point samples. Therefore, the proposed method can be well-combined with existing super-resolution or other interpolation methods. The use of the proposed method with other CFA formats will be one of our further research topics. Furthermore, if we use basis functions other than polynomial basis in the reproducing constraint term, there is much space left for improvement in the approximation accuracy. 

## Figures and Tables

**Table 1:** Comparison of the PSNR between the different methods. The two best values are in bold font.

		McMaster Dataset Images								
Noise Level	Methods	1	2	3	4	5	6	7	8	9
σ=7.65	RI	**25.4925**	**29.7198**	**27.4230**	**28.7656**	**28.7352**	**30.2073**	**30.3072**	**31.3710**	**30.3286**
	ADMM	24.0986	28.7480	25.8632	27.1826	27.4595	28.9430	28.9478	30.1183	29.2356
	BM3D	**25.5562**	**30.3227**	**28.5314**	**30.5665**	**28.9093**	**30.6146**	**31.2559**	**32.7216**	**30.9308**
	Proposed	25.4436	29.5762	27.3741	28.8772	28.6393	29.9254	29.4552	31.4400	30.5131
σ=12.75	RI	24.7643	28.1071	26.3138	27.3294	27.2902	28.3014	28.4306	29.4504	28.4691
	ADMM	23.9313	28.4944	25.7016	26.9503	27.3179	28.7975	28.7118	29.5892	29.0134
	BM3D	**25.0413**	**29.371**2	**27.2120**	**29.2910**	**28.0784**	**29.6043**	**29.7767**	**31.4700**	**29.8660**
	Proposed	**25.0890**	**29.0947**	**26.8136**	**28.2796**	**28.1961**	**29.5057**	**29.1555**	**30.7407**	**29.9819**
		**McMaster Dataset Images**								
**Noise Level**	**Methods**	**10**	**11**	**12**	**13**	**14**	**15**	**16**	**17**	**18**
σ=7.65	RI	**31.4582**	**32.0506**	31.5159	32.7740	32.1560	32.3301	**28.2429**	**27.5546**	**29.3230**
	ADMM	30.2806	31.4607	30.8019	33.7572	32.2113	32.4052	26.7781	25.8872	28.5202
	BM3D	**32.0903**	**32.7758**	**33.0493**	**35.1113**	**33.5730**	**33.4575**	**28.2952**	**27.4109**	**30.2682**
	Proposed	31.2061	31.8866	**32.2402**	**34.4451**	**32.7570**	**32.8573**	27.7978	27.1630	29.1151
σ=12.75	RI	29.2452	29.6592	29.0120	29.6388	29.6244	29.8651	27.0536	26.5489	27.7879
	ADMM	29.8837	30.9067	30.5479	33.4766	31.8245	31.9279	26.5367	25.7544	28.2356
	BM3D	**30.9424**	**31.6128**	**31.7795**	**34.1728**	**32.6172**	**32.4932**	**27.3090**	**26.6519**	**29.2443**
	Proposed	**30.7439**	**31.4394**	**31.4415**	**33.7712**	**32.2474**	**32.3134**	**27.3533**	**26.8395**	**28.6549**

**Table 2:** Comparison of the FSIM between the different methods. The two best values are in bold font.

		McMaster Dataset Images								
Noise Level	Methods	1	2	3	4	5	6	7	8	9
σ=7.65	RI	0.9709	**0.9694**	0.9740	0.9752	0.9724	**0.9710**	**0.9702**	0.9768	0.9700
	ADMM	0.9607	0.9570	0.9637	0.9750	0.9668	0.9599	0.9559	0.9705	0.9665
	BM3D	**0.9820**	**0.9823**	**0.9859**	**0.9859**	**0.9838**	**0.9837**	**0.9829**	**0.9859**	**0.9829**
	Proposed	**0.9732**	0.9672	**0.9774**	**0.9804**	**0.9738**	0.9635	0.9647	**0.9770**	**0.9723**
σ=12.75	RI	0.9618	0.9528	0.9605	0.9493	0.9572	0.9550	0.9511	0.9472	0.9473
	ADMM	0.9599	0.9572	0.9626	**0.9734**	**0.9671**	**0.9610**	0.9567	0.9681	**0.9654**
	BM3D	**0.9709**	**0.9694**	**0.9740**	**0.9752**	**0.9724**	**0.9710**	**0.9702**	**0.9768**	**0.9700**
	Proposed	**0.9663**	**0.9607**	**0.9703**	0.9715	0.9655	0.9561	**0.9582**	**0.9692**	0.9650
		**McMaster Dataset Images**								
**Noise Level**	**Methods**	**10**	**11**	**12**	**13**	**14**	**15**	**16**	**17**	**18**
σ=7.65	RI	**0.9753**	**0.9646**	0.9739	0.9773	**0.9773**	0.9714	**0.9672**	0.9595	**0.9725**
	ADMM	0.9687	0.9606	0.9692	0.9780	0.9759	0.9712	0.9497	0.9538	0.9697
	BM3D	**0.9857**	**0.9795**	**0.9854**	**0.9859**	**0.9857**	**0.9820**	**0.9832**	**0.9776**	**0.9844**
	Proposed	0.9730	0.9615	**0.9779**	**0.9791**	0.9766	**0.9738**	0.9597	**0.9652**	**0.9725**
σ=12.75	RI	0.9491	0.9493	0.9397	0.8805	0.9334	0.9355	**0.9695**	**0.9640**	0.9614
	ADMM	**0.9673**	**0.9598**	0.9685	**0.9768**	**0.9740**	**0.9709**	0.9493	0.9526	**0.9692**
	BM3D	**0.9753**	**0.9646**	**0.9739**	**0.9773**	**0.9773**	**0.9714**	**0.9672**	**0.9595**	**0.9725**
	Proposed	0.9662	0.9533	**0.9706**	0.9719	0.9700	0.9672	0.9537	0.9574	0.9658

**Table 3:** Comparison of the SSIM between the different methods. The two best values are in bold font.

		McMaster Dataset Images								
Noise Level	Methods	1	2	3	4	5	6	7	8	9
σ=7.65	RI	**0.9281**	**0.9163**	0.8833	0.8713	0.9124	**0.8964**	**0.8386**	0.8447	0.9571
	ADMM	0.9026	0.9033	0.8655	0.8743	0.9025	0.8786	0.7760	0.8383	0.9550
	BM3D	**0.9318**	**0.9319**	**0.9222**	**0.9234**	**0.9259**	**0.9067**	**0.8786**	**0.9218**	**0.9674**
	Proposed	0.9245	0.9152	**0.9018**	**0.8995**	**0.9165**	0.8907	0.7963	**0.8897**	**0.9620**
σ=12.75	RI	0.9116	0.8763	0.8234	0.7795	0.8667	0.8475	0.7578	0.7365	0.9293
	ADMM	0.8991	0.8954	0.8541	0.8663	0.8966	0.8730	0.7629	0.7856	0.9497
	BM3D	**0.9203**	**0.9167**	**0.9005**	**0.9032**	**0.9115**	**0.8866**	**0.8298**	**0.9041**	**0.9588**
	Proposed	**0.9188**	**0.9052**	**0.8824**	**0.8758**	**0.9058**	**0.8782**	**0.7844**	**0.8572**	**0.9564**
		**McMaster Dataset Images**								
**Noise Level**	**Methods**	**10**	**11**	**12**	**13**	**14**	**15**	**16**	**17**	**18**
σ=7.65	RI	0.9602	0.9548	0.9833	0.9832	0.9445	0.9613	**0.9495**	**0.9318**	0.9313
	ADMM	0.9644	0.9539	0.9834	0.9874	0.9608	0.9691	0.9346	0.9088	0.9299
	BM3D	**0.9734**	**0.9645**	**0.9894**	**0.9904**	**0.9685**	**0.9747**	**0.9527**	**0.9328**	**0.9496**
	Proposed	**0.9674**	**0.9570**	**0.9868**	**0.9886**	**0.9625**	**0.9703**	0.9449	0.9266	**0.9365**
σ=12.75	RI	0.9312	0.9257	0.9687	0.9660	0.8988	0.9333	0.9266	0.9095	0.8960
	ADMM	0.9576	0.9453	0.9818	0.9864	0.9540	0.9621	0.9276	0.9034	0.9208
	BM3D	**0.9670**	**0.9554**	**0.9859**	**0.9883**	**0.9628**	**0.9697**	**0.9400**	**0.9184**	**0.9370**
	Proposed	**0.9600**	**0.9508**	**0.9842**	**0.9868**	**0.9546**	**0.9644**	**0.9382**	**0.9183**	**0.9262**

## Abbreviations

The following abbreviations are used in this manuscript:

MLS-TV	Moving least squares methods with total variation minimization
RI	Residual interpolation
